# Cognition in Patients with Spinocerebellar Ataxia 1 (SCA1) and 2 (SCA2): A Neurophysiological and Neuropsychological Approach

**DOI:** 10.3390/jcm13164880

**Published:** 2024-08-19

**Authors:** Fabiana Colucci, Sara Stefanelli, Elena Contaldi, Andrea Gozzi, Alessia Marchetti, Maura Pugliatti, Michele Laudisi, Pietro Antenucci, Jay Guido Capone, Daniela Gragnaniello, Mariachiara Sensi

**Affiliations:** 1 Clinical Neurology Unit, Department of Neuroscience and Rehabilitation, University of Ferrara, 44124 Ferrara, Italy; andreagozzi20@gmail.com (A.G.); alessi.marchetti@edu.unife.it (A.M.); pglmra@unife.it (M.P.); ldsmhl@unife.it (M.L.); pietro.antenucci1@gmail.com (P.A.); 2 Parkinson and Movement Disorders Unit, Department of Clinical Neurosciences, Fondazione IRCCS, Istituto Neurologico Carlo Besta, 20133 Milan, Italy; 3 Neurology Unit, Department of Neuroscience, Azienda Ospedaliero-Universitaria S. Anna, 44124 Ferrara, Italy; stefanelli.psicologa@gmail.com (S.S.); jaycap71@gmail.com (J.G.C.); d.gragnaniello@ospfe.it (D.G.); mchiasen@gmail.com (M.S.); 4 Centro Parkinson e Parkinsonismi, ASST Gaetano Pini-CTO, 20122 Milan, Italy; contaldie@yahoo.it

**Keywords:** spinocerebellar ataxia, SCA1, SCA2, auditory event potential, ERP, cognition, cognitive decline

## Abstract

**Background/Objectives**: Cognitive impairment in spinocerebellar ataxia patients has been reported since the early-disease stage. We aimed to assess cognitive differences in SCA1 and SCA2 patients. **Methods**: We performed neuropsychological (NPS) and neurophysiological (auditory event-related potentials, aERPs) assessments in 16 SCA1 and 18 SCA2 consecutive patients. Furthermore, clinical information (age at onset, disease duration, motor disability) was collected. **Results**: NPS tests yielded scores in the normal range in both groups but with lower scores in the Frontal Assessment Battery (*p* < 0.05) and Visual Analogue Test for Anosognosia for motor impairment (*p* < 0.05) in SCA1, and the Trail Making Test (*p* < 0.01), Raven’s progressive matrices (*p* < 0.01), Stroop (*p* < 0.05), and emotion attribution tests (*p* < 0.05) in SCA2. aERPs showed lower N100 amplitude (*p* < 0.01) and prolonged N200 latency (*p* < 0.01) in SCA1 compared with SCA2. Clinically, SCA2 had more severe motor disability than SCA1 in the Assessment and Rating of Ataxia Scale. **Conclusions**: SCA2 showed more significant difficulties in attentional, visuospatial, and emotional function, and greater motor impairment. In contrast, SCA1 showed less cognitive flexibility/phasic ability, probably affected by a more severe degree of dysarthria. The same group revealed less neural activity during nonconscious attentional processing (N100-N200 data), suggesting greater involvement of sensory pathways in discriminating auditory stimuli. NFS did not correlate with NPS findings, implying an independent relationship. However, the specific role of the cerebellum and cerebellar symptoms in NPS test results deserves more focus.

## 1. Introduction

Spinocerebellar ataxias (SCAs) are a group of rare neurodegenerative disorders with autosomal dominant inheritance. The prevalence of SCA is estimated at 2–3 per 100,000 individuals [1]. In Italy, SCA1 and SCA2 are the most frequently diagnosed types [2,3] and are linked to polyglutamine repeat-expansion forms in *ATXN1* and *ATXN2* genes, which become pathological when expansion is greater than 42 and 35, respectively [3]. The neurodegeneration in cerebellar neurons leads to motor deficits and cerebellar ataxia is the prominent symptom, but neurodegeneration might involve other parts of the central and peripheral nervous systems, leading to heterogeneous symptoms. Indeed, in addition to cerebellar motor impairment, cognitive, behavioral, and affective functions may also be affected [4].

However, how the different types of cerebellar degeneration act on cognitive processes is still debated. Several studies have assessed the clinical cognitive/affective differences between SCA1 patients and SCA2 patients, but the results are still controversial [5,6,7,8,9,10,11]. The brain synaptic function during cognitive processes could be electrophysiologically studied through the auditory event-related potential (aERP). In particular, the ERP P300 component has been widely used to study processes such as attention, discrimination, and working memory; thus, the P300 could reflect cognitive decline related not only to aging, but also to some neurodegenerative pictures as found in Alzheimer’s disease [12,13,14,15]. In cerebellar ataxia, such as SCA1 and SCA2, aERPs have been studied for this purpose [16,17,18].

To the best of our knowledge, no studies have compared the cognitive outcome based on auditory event-related potential in these two diseases.

We aimed to assess the differences in cognition between SCA1 and SCA2 through clinical and neurophysiological examination and in relation to demographics, motor clinical features, and disease duration.

## 2. Materials and Methods

This monocentric, cross-sectional observational cohort study was conducted at the University Hospital of Ferrara between 2021 and 2023.

### 2.1. Participants

Genetically confirmed SCA1 or SCA2 patients, referred to the Movement Disorders Centre of Ferrara Hospital, were consecutively screened for eligibility criteria between July 2021 and December 2023. Thirty-four patients, 16 SCA1 and 18 SCA2, were enrolled. Patients with severe cerebellar impairment [i.e., motor Scale for Assessment and Rating of Ataxia (SARA) score > 24] and/or cognitive impairment and/or a history of hearing loss, were excluded. All participants were Italian native speakers and were able to provide informed consent.

None of the participants were biologically related except two sisters in the SCA1 group.

The study protocol was approved by the local institutional review board (CE 453/2021), and all participants gave informed written consent. The study conformed with the ethical standards stated in the 1964 Declaration of Helsinki and its later amendments.

### 2.2. Data Collection

#### Clinical Data

We collected data on age at onset of motor cerebellar symptoms, disease duration at the time of enrollment, number of CAG repeat-expansions, comorbidities, therapies, and paternal or maternal inheritance. In addition, disease motor severity was assessed through the SARA (scores from 0 to 40) [19].

All the data from the SCA1 population were already reported in a previous publication of our group [16].

### 2.3. Neuropsychological Testing

An extensive neuropsychological battery was administered in about an hour and a half in a quiet hospital room. Psychometric evaluations were performed by the same neuropsychologist (SS). Raw scores were adjusted to Italian normative data for sex, age, and education, and available cut-off scores were used to define an abnormal test performance. During the preliminary interview, no significant psychiatric disorders or mood alterations were noted. The battery included the following tests.

- Mini-mental State Examination (MMSE): Test for brief screening of the cognitive status, where a score below the cut-off of 24 indicates cognitive impairment [20].
- Frontal Assessment Battery (FAB): Widely used to assess executive functions. It consists of 6 subtests exploring conceptualization, mental flexibility, motor programming, sensitivity to interference, inhibitory control (go–no-go), and environmental autonomy [21].
- Verbal fluency test (F-A-S letters): This a lexical retrieval test in which the subject is invited to produce as many words as possible using phonemic criteria in one minute; it requires processing speed, and is considered to assess executive functions [22].
- Trail Making Test (TMT) A-B: In the TMT-A, it is required to connect twenty-five numbered circles with a direct line in ascending order; this part assesses selective attention and motor speed. In TMT-B, it is required to alternate thirteen numbers and letters, also implicating attentional shifting. The B-A score is considered as a measure of executive functions [23].
- Raven Colored Progressive Matrices (RCPMs): In this test, it is required to choose, from six items, the missing element that completes a matrix 2 × 2. It assesses non-verbal reasoning ability and visuospatial processing skills [24].
- Stroop Test: This consists of three parts which demand word reading, color naming, and color–word reading. This involves many cognitive functions such as selective attention, sensitivity to interference, and inhibitory control; thus, it is also considered a test for executive functions [25].
- Rey–Osterrieth Complex Figure (ROCF): This consists of two different parts: the first investigates the ability of construction practice and visuospatial planning, and the second explores the visual memory by the recall of the model after fifteen minutes [26].
- Prose memory test (Babcock’s short tale—BST): A short story has to be stored and recalled after ten minutes. This test measures the verbal-episodic memory [27].
- Emotion Attribution Task (EAT): This test demands the recognition of the emotions of a subject in 58 short stories which can elicit basic emotions (i.e., happiness, sadness, anger, fear, envy, embarrassment, and disgust). The test is considered to assess social cognition [28].
- Visual Analogue Test for Anosognosia for motor impairment (VATA-m): A questionnaire that compares patient self-evaluation to his/her caregiver’s evaluation of the patient’s abilities in a series of motor tasks [29].

### 2.4. Electrophysiological Assessment

KeypointTM software (Natus Neurology Incorporated, Middleton, WI, USA) was used to deliver aERPs using the oddball paradigm. The paradigm included at least 100 “standard” and “target” auditory stimuli presented pseudo-randomly through earphones (mean sound level of 74.97 ± 3.15 dB; frequency of 2000 Hz for standard stimuli with 80% presentation probability, and 1500 Hz for target stimuli with 20% presentation probability; stimuli duration of 200 ms, inter-stimulus interval 1200 ms). Participants were instructed to focus on the target stimuli and perform a simple motor task upon their onset (e.g., clicking a pen).

EEG signals were recorded from scalp electrodes at Fz, Cz, and Pz sites (international 10/20 system), referencing linked earlobe electrodes [16,30]. Peak latency and amplitude of N100, N200, and P300 components were measured in response to target tones at the Cz electrode.

For data analysis, we focused on the P3b subcomponent of the P300, which reflects attentional allocation and memory updating [31]. P300 amplitude reflects the brain activity required to maintain working memory during stimulus context updating and information processing; in fact, lower P300 amplitude is associated with aging and lower performance in memory, attention, and executive functions. P300 latency reflects neural speed or brain efficiency and is considered an index of the processing time required before response generation.

We also focused on the early components of ERPs, N100 and N200, which represent an automatic process of novelty detection as attentional abilities at the non-conscious level [16,32]. Indeed, N100 reflects neural processes sensitive to stimulus features such as the abruptness of the onset and end of sound, and N200 is evoked before the motor response, suggesting its link to cognitive processes of stimulus identification and distinction, generated independently of attention to stimuli [16,32].

Artefact rejection criteria were applied to ensure data quality. Amplitude was determined using the baseline-to-peak method.

### 2.5. Statistical Analysis

Categorical variables are presented in counts or percentages while mean ± standard deviation (SD) or median and interquartile range (IQR) are used for continuous variables. The comparison between binary variables was assessed using the chi-square test or Fisher’s exact test. In contrast, differences between continuous variables were analyzed using the *t*-test or the Mann–Whitney test according to variable distribution. The Spearman rho test was employed to explore correlations between continuous variables. The assessment of the differences between groups was obtained by analyses of variance (ANOVA) and covariance (ANCOVA). Statistical analyses were conducted with SPSS software support (IBM (Armonk, NY, USA), v20), and statistical significance was assigned for results of *p* < 0.05.

## 3. Results

The study was conducted on sixteen SCA1 (nine men and seven women) and eighteen SCA2 (eight men and ten women). The mean age (SD) at the time of the study was 47.69 (8.16) [47.8 (6.9) and 48.1 (10.2) years for men and women, respectively (*p* = 0.933)] in SCA1, and 44.7 (12.47) [43.9 (12.7) and 46.0 (13.0) years in men and women (*p* = 0.732)] in SCA2. All patients, except one, were right-handed, and the average level of schooling was 12.5 (3.03) years for SCA1 and 12.6 (2.3) years for SCA2. The age of onset of cerebellar first symptom in SCA1 was 41.27 (8.50) years, with a mean disease duration of 6.47 (3.62) years; in SCA2, the age of onset was 35.61 (10.49) years, with a mean duration of 9.44 (5.87) years (age at onset *p* < 0.05; disease duration *p* = 0.06). Demographic data and standard deviations (SD) are reported in Table 1.

Table 2 reports mean and standard deviation (SD) of the SARA motor scale and neuropsychological test values by SCA type (SCA1 and SCA2). On average, all patients presented mild-to-moderate motor impairment at the SARA motor scale at the time of evaluation: 11.53 (5.14) in SCA1 and 14.44 (9.26) in SCA2 with no statistically significative difference (*p* = ns). Corrected for disease duration (b) or age at the time of the study and disease duration (c), SCA2 presented a significantly higher score and, therefore, higher disability [(b) SCA1 12.59 (1.74) vs. SCA2 13.50 (1.64), *p* < 0.05; (c) SCA1 12.49 (1.8) vs. SCA2 13.59 (1.7), *p* < 0.05].

**Figure 1:**
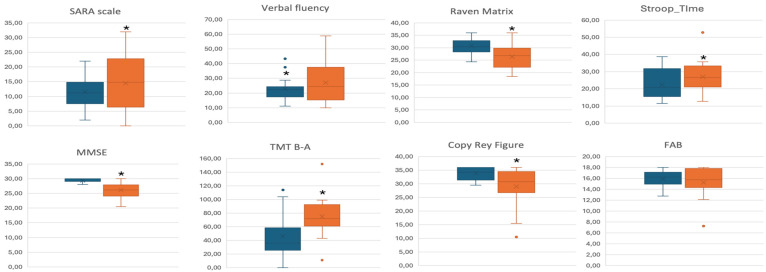
Distribution of SARA and neuropsychological test data by SCA types (SCA1 in blue, SCA2 in orange). Data used for neuropsychological tests are raw scores adjusted for sex, age and education. The median is the center of the box, 25th percentile is the bottom of the box, 75th percentile is the top of the box. Dots represent outlier values. *: statistically significant difference at *p* < 0.05.

All neuropsychological tests were within a normal range in both groups, except for TMT part A in SCA2 [107 (59.11), pathological cut-off higher than 94]. Comparing the two populations, SCA2 performed worse on MMSE, TMT A and B, Raven’s matrices test, Rey’s figure copy, and VATA-m. When analyses were adjusted for disease duration and age at the time of the study, SCA2 also showed a worse performance in the Stroop Test (time) and emotions test, while SCA1 showed a worse performance only in verbal fluency than SCA2 (Figure 1).

Table 3 shows the neurophysiological results of aERPs. No differences between the two SCA populations examined were detected in P300 records regarding both latency and amplitude. Conversely, SCA1, compared to SCA2, had significantly longer N200 latency and lower N100 amplitude (adjusted for disease duration and age).

Table 4 shows bivariate correlations measured by Pearson’s coefficient in SCA2; a direct correlation is observed between disease duration and (i) P300 latency (r = 0.736, *p* = 0.0001), (ii) SARA score (r = 0.528, *p* = 0.024), and (iii) TMT-A (r = 0.576, *p* = 0.020); in addition, inverse correlations between disease duration and (a) P300 amplitude (r = −0.540, *p* = 0.021), (b) N200 amplitude (r = −0–688, *p* = 0.002), (c) FAB (r = −0.548, *p* = 0.028), and (d) MMSE score (r = −0.517, *p* = 0.040) and emotion test (r = −0.778, *p* < 0.001) were also detected.

Regarding N200, the latency is directly correlated to the SARA scale (r = 0.542, p = 0.020); conversely, the amplitude shows a direct correlation to MMSE (r = 0.707, *p* = 0.002) and FAB (r = 0.520, *p* = 0.039), and an inverse correlation to VATAm (r = −0.791, *p* = <0.001). The latency of N100 directly correlates to TMT-B (r = 0.627, *p* = 0.029), while the N100 amplitude correlates to emotion test (r = 0.522, *p* = 0.038). A negative trend between N100 amplitude and disease duration was also detected (r = −0.468, *p* = 0.050).

The SARA scale has a direct correlation not only with disease duration (r = 0.528, *p* = 0.024), but also with TMT-A (r = 0.656, *p* = 0.006), and an inverse correlation with the Copy of Rey Figure (r = −0.621, *p* = 0.010).

Finally, a trend toward an inverse correlation between SARA and FAB is also observed (r = −0.495, *p* = 0.051) (Table 4).

In SCA1, the bivariate correlations measured by Pearson’s coefficient between neurophysiological outcomes and aERP and motor severity are described in a previous article [16]. Briefly, significant inverse correlations were found between P300 latency and EAT scores (r = − 0.633, *p* = 0.027). N200 latency inversely correlated with FAB score (r = − 0.520, *p* = 0.047) and directly correlated with Stroop Test (r = 0.538, *p* = 0.039).

## 4. Discussion

In recent years, the cerebellar contribution to cognitive, social, and emotional functions has been widely highlighted in the literature [33,34,35,36,37,38,39,40,41,42]. Evidence from several studies on both healthy individuals and patients with cerebellar damage confirms that those with isolated cerebellar pathology may experience significant cognitive and emotional impairments [33,43,44]. Schmahmann and Sherman, in 1998, through anatomical, physiological, and functional neuroimaging studies, described a “cerebellar cognitive affective syndrome” (CCAS) in 20 patients with diseases restricted to the cerebellum, characterized by impairment of executive function, personality change (such as disinhibited or inappropriate behavior), emotional regulation, and linguistic difficulties [33]. The authors hypothesized the cerebellum participation in the organization of higher-order functions (executive functions, spatial cognition, personality, and language) by the modulation of neural circuits that link the prefrontal, posterior parietal, superior temporal, and limbic cortices with the cerebellum [33,39]. Indeed, neuroimaging studies have revealed that the cerebellum interacts with attentional networks, particularly the ventral network, influencing attentional processes. From an anatomical and functional perspective, the cognitive/limbic area of the cerebellum is found in the posterior lobe [42,45]. Several studies report that damage to the cerebellum, mainly the left cerebellum Crus I and VI lobules, with the subsequent disconnection of the left thalamic projection and left fronto-striatal fascicle, results in poor cognitive processing of understanding, generating, and regulating social behavior (Theory of Mind—ToM), leading to the inability to perform emotional attribution and understand the mental states of others [39,44,45]. In SCA1 and SCA2, atrophy of the brainstem (mainly of the pons) may consequently alter cerebro-cerebellar circuits [5,6,7,46,47], which, in addition to supratentorial atrophy, may explain the associated cognitive decline in SCA [46,47].

Previous works suggest different rates of cognitive impairment in SCA patients [4,9,10,11,12,13,14,15,16,48], including SCA1 and SCA2, but data are scarce and sometimes controversial.

Comparing the two more common types of SCA in Italy, SCA1 and SCA2, our study confirmed a more consistent and widespread cognitive involvement in SCA2 (i.e., MMSE) [4,9,10,11,48]. Indeed, SCA2 showed worse performance in several core attentional functions, such as sustained (TMT-A, TMT-B) and selective attention (TMT-A, Stroop Test time), visual-perceptual skills (Raven’s matrices test, Copy Rey Figure), and executive functions (Stroop Test time), confirming some literature findings [8,9,10,11,48].

Exploring cognition in SCA could be difficult considering the variable role of motor accuracy and motor speed, which are both required in many psychometric tests. Fancellu et al. [10], for example, found cognitive deficits compared to controls in phonemic and semantic fluencies, and attentional matrices in both SCA1 and SCA2, but they did not show alterations in the modified-Wisconsin Card Sorting Test (mWCST). The authors explained this normal outcome considering that mWCST evaluates executive functions such as planning and monitoring, without requiring psychomotor or articulatory skills usually impaired in cerebellar patients. Conversely, for Klinke et al., neuropsychological impairment of associated frontal functions is independent of motor disabilities in SCA1, 2, and 3 patients, but depends on disruption of basal ganglia-thalamocortical loops [7].

In light of the motor impairment, in our study group, SCA2 patients had greater motor severity and disabilities on the SARA scale than SCA1 patients; this might influence our results [9].

In our previous work [16] on SCA1, significant correlations between the severity of cerebellar motor involvement and TMT-A/B (direct) were observed. Similarly, in this work, SCA2 patients with higher SARA scores showed higher TMT-A and lower Rey Copy Figure scores. Instead, higher motor impairment correlated with a lower performance in Raven’s Matrices test, which requires any motor skills, only in SCA1 [16], but not in SCA2 patients. These data suggest that, in SCA2, the neuropsychological tests, which do not need motor tasks, unlike the Raven’s Matrices test, are better performed. This confirms the results of Gigante et al., who hypothesized a possible negative influence of motor disabilities on executive functions outcomes in SCA2 patients [9].

Ma et al. also detected a positive correlation between cognitive impairments and clinical severity of ataxia symptoms in patients with SCA 1, 2, and 3, among whom SCA2 had more severe ataxia [8]. However, they found similar impairment between groups, except for verbal fluency and word memory dysfunction, which were higher in SCA2 and SCA3. Our findings did not support the results of Ma et al., because we detected similar verbal memory (prose memory test) in SCA1 and SCA2, confirming what has already been reported in other literature studies [4,5,6,7]. We also documented lower scores in phonemic verbal fluency in SCA1, albeit in the normal range, documenting a slightly lower degree of cognitive flexibility/phasic ability. However, we did not evaluate and correct these data on the grade of dysarthria, as suggested in a recent acoustic analysis study, where SCA1 patients showed slowed speech and longer single-syllable pronunciation duration during speech [49].

Regarding social cognition, mainly emotion attribution, deficits have been reported in SCA [4,42,50,51]. Tamas et al., detected a more pronounced impairment in SCA1 and SCA2 compared to idiopathic late-onset cerebellar ataxia (ILOCA) patients [42]; however, the authors did not compare the two types of SCA [42]. A comparison of the social cognition in the two SCA types was analyzed by Sokolovsky et al., who reported a more pronounced deficit in emotion attribution in two SCA1 compared to three SCA2 [4]. The present work did not confirm the results of Sokolovsky et al., revealing a lower ability in emotion attribution in SCA2 compared to SCA1. We believe that, although we used a different test, our data were more reliable, analyzing a larger number of patients (18 SCA2 and 16 SCA1) and supporting the results of the critical review of Giocondo and Curcio, who reported normal performance in SCA1 in emotion attribution tasks, while in SCA2 the social cognitive profile was characterized by a very low performance in the same tasks [51].

Concerning motor self-awareness, to the best of our knowledge, our work is the first one that uses VATA-m to assess anosognosia in SCA, detecting a higher degree of anosognosia in SCA1 compared to SCA2. Anosognosia, classically traced to lesions of the right parietal, frontal, temporoparietal, thalamic, or basal ganglia lobes [52,53,54], may be the consequence of greater cerebellar degeneration in SCA1 [4,40].

In our study, the relation between neurophysiological and cognitive evaluation was investigated with aERPs. aERP alterations (increased P300 latency and decreased P300 amplitude) were described in SCA1 and SCA2 patients, which might precede the clinical onset of cerebellar manifestations and are closely associated with the progression of cerebellar disorders [16,17,18,55].Therefore, P300 abnormalities could represent a potential pre-clinical biomarker of the disease [56] and an electrophysiological marker for identifying early cognitive dysfunction in SCA [16,17,18]. It can be hypothesized that cerebellar involvement in SCA1, as in SCA2, may alter P300 components by interfering with attentional processes due to the dysfunction of cerebellar projections to the prefrontal and posterior-parietal cortices, which are involved in cognitive processes [57].

These abnormalities have been described to be a function of both longer disease duration and motor severity [17]. In our previous data on SCA1, we found a correlation of P300 changes (increased latency and decreased amplitude) with motor severity. Instead, in the SCA2 population, we found a correlation with disease duration. Similar to Rodriguez et al., in SCA2 [17] and SCA1 [16], we did not find any correlation in latency and amplitude of P300 with a single neuropsychological test administered to investigate the executive function.

Differently from Rodriguez et al. [17], we also analyzed the early components of ERPs, N100 and N200, in the SCA2 population, as we did in our previous work on the SCA1 population [16]. N100 reflects neural processes sensitive to stimulus features (e.g., the abruptness of the onset and end of sound), and has been reported to be altered in cerebellar [58] and non-cerebellar diseases such as Parkinson’s disease (PD) [59] and Alzheimer’s disease (AD) [15]. From a neuropsychological point of view, it has been related to the visual working memory process [59]. Contrary to our previous works on SCA1, in the present work on SCA2, N100 latency has been linked to executive function, but mainly to the attentional shift process (TMT-B).

Regarding the second early component of aERP, N200, it is evoked before the conscious response, suggesting its link to cognitive processes of stimulus identification and distinction, generated independently of attention to stimuli. N200 wave has been hypothesized to be a potential biomarker for cognitive decline in MCI, preclinical AD [15], and PD [59], and is mainly altered during semantic discrimination tasks [60]. In cerebellar patients, Yang et al. did not find abnormalities of N200 in X-Fragile ataxia patients [58]; conversely, in our previous study on SCA1, N200 was linked to executive functions (FAB) [9], and the present work on SCA2 confirmed the same correlation.

Comparing the outcome of aERPs in these two populations, no significant differences in P300 latency and amplitude were found. A lower amplitude of the N100 and a prolonged latency of the N200 were, however, observed in SCA1 compared with SCA2, and statistical significance was maintained even after adjusting for disease duration and age of the patients at the time of the study. These results on N100 and N200 data would suggest an involvement of sensory pathways in the discrimination and categorization of auditory stimuli in this group of patients. Indeed, SCA1, compared with SCA2, might have a greater auditory deficit peripherally at brainstem aERP level [5] and a mild-to-moderate demyelinating neuropathy [61]. This peripheral involvement could explain the difficulty in recognizing stimuli in the periphery and the possible substrate of the non-conscious attentional processes’ alteration.

The limitations of the present study are the unavailability of a group of healthy controls to compare with SCA2 population data; the lack of neuroimaging features that could have helped to correlate anatomy to cognitive function; the use of a neuropsychological battery with several ataxia-dependent tests; and the cross-sectional design and the relatively small sample size, which requires a cautious interpretation of our findings. In addition, to assess social cognition, compared to the literature, we investigated only one of the components, emotion attribution. Another missing point is the lack of a quantitative neuropsychiatric assessment through specific questionnaires investigating personality or behavior, and/or depression/anxiety.

## 5. Conclusions

Despite an almost normal cognitive profile in all participants, SCA2 showed worse global cognitive function values with MMSE and lower performance in attentional and visuospatial processes (TMT-A, TMT-B, Raven’s matrices, Copy Rey Figure).

The neurophysiological evaluation, instead, did not show significative differences regarding the conscious component of P300 among the two populations of SCA1 and SCA2, while a significative difference was documented in N100 and N200, the non-conscious component, which was found to be worse in SCA1.

In addition, no correlation between P300 and the neuropsychological test was found in the SCA2 population, confirming our previous data on SCA1. On the other hand, regarding the early aERP components, in both SCA populations, N200 was correlated with executive function (FAB), while N100 was correlated with the attentional shift process only in SCA2.

In conclusion, the slight differences in aERP early components between the two populations, SCA1 and SCA2, did not reflect the differences in the neuropsychological assessment; therefore, they cannot be considered useful biomarkers to distinguish different levels of cognitive involvement between the two populations. The neuropsychological features are more consistent in detecting and characterizing cognitive differences in these patients.

Future works are needed to better understand the utility of this neurophysiological tool in discriminating different forms of SCA.

## Figures and Tables

**Table 1:** Demographic characteristics of SCA1 and SCA2 participants.

	SCA1	SCA2	*p*
Number of participants	16	18	
Sex (males/females)	9/7	8/10	
Age (years), mean (SD)	47.69 (8.16)	44.77 (12.47)	*ns*
Educational level (years), mean (SD)	12.5 (3.03)	12.6 (2.3)	*ns*
Disease duration (years), mean (SD)	6.47 (3.62)	9.44 (5.87)	*=0.06*
Age at onset (years), mean (SD)	41.27 (8.50)	35.61 (10.49)	***<0.05***
Parental inheritance (Paternal/maternal)	4/12	6/12	
Number of repeats expanded allele, mean (SD)	47.73 (6.44)	40.5 (5.16)	
Symptom onset (N)			
- Unsteadiness	13	10	
- Dysarthria	2	2	
- Stiff legs	1	0	
- Cramps/pain	0	6	
Comorbidities (N)			
- Diabetes	0	1	
- Hypertension	1	2	
- Migraine	1	0	
- Gastroesophageal reflux	1	2	
- Cancer	1 (Ovarian)	0	
- Autoimmune disease	1 (Hashimoto thyroiditis)	0	

Parametric data are presented as mean and standard deviations (SD) in brackets; categorical data are presented as numbers. In bold, statistically significant difference at *p* < 0.05; *ns*: no statistically significant difference at *p* < 0.05.

**Table 2:** SARA scale and neuropsychological test data by SCA type (SCA1 and SCA2).

Test (Pathological Cut-Off)	SCA1 ^a^	SCA2 ^a^	*p* ^a^	SCA1 ^b^	SCA2 ^b^	*p* ^b^	SCA1 ^c^	SCA2 ^c^	*p* ^c^
**SARA**	11.53 (5.13)	14.44 (9.26)	*ns*	12.59 (1.74)	13.50 (1.64)	***<0.05***	12.49 (1.80)	13.59 (1.70)	***<0.05***
**MMSE (<24)**	29.33 (0.82)	26.16 (2.80)	***<0.01***	29.11 (0.52)	26.37 (0.50)	***<0.01***	29.01 (0.52)	26.47 (0.50)	***<0.01***
**FAB (<13.4)**	15.79 (1.37)	15.23 (2.79)	*ns*	15.58 (0.53)	15.50 (0.52)	*ns*	15.40 (0.51)	15.69 (0.50)	*ns*
**Verbal Fluency (<17.3)**	22.90 (8.46)	27.09 (8.46)	*ns*	22.58 (3.00)	27.40 (2.90)	*ns*	21.23 (2.70)	28.65 (2.61)	***<0.05***
**TMT A (>94)**	68.93 (28.04)	116.31 (58.16)	***=0.01***	75.98 (11.50)	110.14 (10.72)	***<0.01***	74.15 (11.71)	102.79 (12.10)	***<0.05***
**TMT B (>187)**	121.08 (37.0)	190.42 (65.30)	***<0.01***	123.51 (14.31)	187.78 (14.90)	***<0.01***	125.90 (13.64)	169.23 (14.93)	***<0.01***
**TMT B_A (>187)**	45.93 (31.14)	74.92 (33.84)	***<0.05***	45.66 (8.85)	75.23 (9.56)	***<0.01***	48.19 (6.66)	71.47 (12.20)	***<0.01***
**Stroop Test Time (>36.92)**	21.57 (8.52)	26.94 (9.76)	*ns*	22.56 (2.59)	26.84 (2.41)	*ns*	23.75 (2.32)	25.81 (2.16)	***<0.05***
**Raven’s Matrices (<18.96)**	30.75 (3.29)	26.44 (4.86)	***<0.01***	30.55 (1.10)	26.52 (1.06)	***<0.05***	30.02 (0.96)	27.12 (0.93)	***<0.01***
**Rey’s Figure Copy (<28.53)**	33.79 (2.43)	28.97 (7.37)	***<0.05***	32.85 (1.47)	29.74 (1.32)	***<0.01***	33.09 (1.53)	29.53 (1.37)	***<0.05***
**Rey’s Figure Recall (<9.46)**	13.16 (5.47)	10.57 (5.64)	*ns*	13.07 (1.60)	10.64 (1.49)	*ns*	12.17 (1.50)	11.43 (1.29)	*ns*
**Prose memory test (Babcock’s tale) (<8.2)**	8.87 (3.37)	12.45 (14.54)	*ns*	8.11 (2.76)	13.16 (2.67)	*ns*	7.97 (2.85)	13.30 (2.75)	*ns*
**Emotions Test (<44.19)**	48.83 (8.49)	44.25 (7.80)	*ns*	47.82 (2.22)	45.01 (1.91)	***<0.05***	47.89 (2.29)	44.96 (1.97)	***<0.05***
**VATA-m**	6.00 (5.16)	1.60 (3.25)	***<0.05***	6.20 (1.23)	1.43 (1.14)	***<0.05***	6.03 (1.25)	1.58 (1.15)	***=0.057***

Parametric data are presented as mean and standard deviation (SD). In bold statistically significant difference at *p* < 0.05; *ns*: no statistically significant difference at *p* < 0.05. ^a^ Mean and standard deviation (SD) obtained with Student’s *t*-test. ^b^ Marginal means and standard deviation (SD) obtained with ANCOVA, corrected for duration of illness at time of study. ^c^ Marginal means and standard deviation (SD) calculated with ANCOVA, corrected for disease duration and age at the time of study.

**Table 3:** Neurophysiological data by SCA types (SCA1 and SCA2).

	SCA1 ^a^	SCA2 ^a^	*p* ^a^	SCA1 ^b^	SCA2 ^b^	*p* ^b^	SCA1 ^c^	SCA2 ^c^	*p* ^c^
P300									
Latency	390.18 (58.32)	386.11 (37.94)	*0.814*	394.82 (11.94)	381.99 (1.23)	*0.179*	393.82 (12.40)	382.88 (11.63)	*0.318*
Amplitude	3.99 (4.36)	4.26 (3.78)	*0.849*	4.06 (1.03)	4.19 (0.97)	*0.699*	3.48 (0.98)	4.71 (0.91)	*0.073*
N100									
Latency	101.32 (6.32)	101.78 (11.31)	*0.887*	101.31 (2.37)	101.78 (2.24)	*0.990*	101.45 (2.48)	101.66 (2.33)	*0.994*
Amplitude	6.04 (2.51)	7.05 (3.24)	*0.324*	6.18 (0.68)	6.92 (0.64)	***0.043***	5.66 (0.58)	7.38 (0.55)	***0.0003***
N200									
Latency	260.25 (41.32)	219.61 (19.30)	***0.002***	261.18 (7.82)	218.79 (7.37)	***0.001***	263.49 (7.96)	216.73 (7.48)	***0.002***
Amplitude	3.61 (1.93)	4.94 (3.19)	*0.155*	3.66 (0.67)	4.90 (0.63)	*0.239*	3.42 (0.68)	5.11 (0.64)	*0.163*

Parametric data are presented as mean and standard deviation (SD). In bold, statistically significant difference at *p* < 0.05; *ns:* no statistically significant difference at *p* < 0.05. ^a^ Mean and standard deviation (SD) calculated through the *t*-test. ^b^ Marginal means and standard deviation (SD) calculated with ANCOVA, corrected for the duration of illness at the time of study. ^c^ Marginal means and standard deviation (SD) calculated with ANCOVA, corrected for the duration of illness and age at the time of study.

**Table 4:** Correlation between neuropsychological, SARA scale, disease duration, and neurophysiological outcomes in SCA2.

		P300 Latency	P300 Amplitude	N200 Latency	N200 Amplitude	N100 Latency	N100 Amplitude	SARA	Disease Duration
**MMSE**	**Pearson’s Coefficient**	−0.171	0.047	0.322	0.707	0.026	0.169	−0.370	−0.517
	***Sig.***	*0.527*	*0.864*	*0.224*	***0.002***	*0.924*	*0.532*	*0.158*	***0.040***
**FAB**	**Pearson’s Coefficient**	−0.029	0.144	−0.086	0.520	0.106	0.346	−0.495	−0.548
	***Sig.***	*0.916*	*0.593*	*0.750*	***0.039***	*0.696*	*0.189*	*0.051*	***0.028***
**Verbal Fluency**	**Pearson’s Coefficient**	0.094	−0.201	−0.274	−0.171	−0.030	0.093	−0.337	−0.100
	***Sig.***	*0.729*	*0.456*	*0.305*	*0.527*	*0.911*	*0.731*	*0.202*	*0.713*
**Stroop_Time**	**Pearson’s Coefficient**	−0.149	0.056	0.119	0.495	−0.076	−0.221	−0.224	−0.066
	***Sig.***	*0.581*	*0.837*	*0.661*	*0.051*	*0.781*	*0.411*	*0.404*	*0.808*
**Raven_Matrix**	**Pearson’s Coefficient**	0.070	0.047	−0.130	0.272	−0.265	−0.057	−0.270	−0.244
	***Sig.***	*0.796*	*0.862*	*0.632*	*0.308*	*0.321*	*0.834*	*0.312*	*0.362*
**Copy_Rey_** **Figure**	**Pearson’s Coefficient**	−0.160	0.412	−0.367	0.192	−0.060	0.317	−0.621	−0.486
	***Sig.***	*0.554*	*0.113*	*0.162*	*0.492*	*0.826*	*0.231*	***0.010***	*0.056*
**Recall_Rey_** **Figure**	**Pearson’s Coefficient**	0.310	−0.243	−0.104	0.029	−0.021	−0.131	−0.044	0.176
	***Sig.***	*0.262*	*0.382*	*0.711*	*0.916*	*0.940*	*0.643*	*0.877*	*0.531*
**Prose_Memory_Test**	**Pearson’s Coefficient**	−0.424	0.605	−0.296	0.493	−0.021	0.368	−0.400	−0.383
	***Sig.***	*0.102*	*0.053*	*0.266*	*0.053*	*0.937*	*0.161*	*0.125*	*0.143*
**Emotion_test**	**Pearson’s Coefficient**	−0.376	0.472	0.077	−0.119	0.000	0.522	−0.355	−0.778
	***Sig.***	*0.151*	*0.065*	*0.776*	*0.673*	*1.000*	***0.038***	*0.177*	***<0.001***
**VATAm**	**Pearson’s Coefficient**	0.155	−0.190	−0.212	−0.791	0.341	0.130	0.193	0.295
	***Sig.***	*0.582*	*0.497*	*0.449*	***<0.001***	*0.214*	*0.644*	*0.492*	*0.286*
**TMT_A**	**Pearson’s Coefficient**	0.228	−0.329	0.215	−0.143	0.373	−0.065	0.656	0.579
	***Sig.***	*0.395*	*0.213*	*0.423*	*0.598*	*0.155*	*0.811*	***0.006***	***0.019***
**TMT_B**	**Pearson’s Coefficient**	−0.091	0.047	0.225	0.237	0.627	0.363	0.474	0.225
	***Sig.***	*0.780*	*0.885*	*0.482*	*0.458*	***0.029***	*0.246*	*0.119*	*0.481*
**TMT_B-A**	**Pearson’s Coefficient**	0.010	0.429	−0.274	0.351	0.365	0.037	−0.088	0.115
	***Sig.***	*0.976*	*0.164*	*0.388*	*0.263*	*0.243*	*0.909*	*0.787*	*0.722*
**SARA**	**Pearson’s Coefficient**	0.308	−0.275	0.541	0.037	0.007	−0.142		0.528
	***Sig.***	*0.214*	*0.270*	***0.020***	*0.883*	*0.978*	*0.575*		***0.024***
**Disease_** **Duration**	**Pearson’s Coefficient**	0.736	−0.540	0.124	−0.688	−0.033	−0.468	0.528	
	***Sig.***	***0.0001***	***0.021***	*0.624*	***0.002***	*0.898*	*0.050*	***0.024***	

Bivariate correlations are measured by Pearson’s coefficient. Significance (Sig.) is reported for each correlation. In bold, statistically significant difference at *p* < 0.05.

## Data Availability

Anonymized data can be obtained upon reasonable request from qualified researchers.
